# Supplementary material for: Intrinsic timescales as an organizational principle of neural processing across the whole rhesus macaque brain
Source: eLife. 2022 Mar 2;11:e75540. doi: 10.7554/eLife.75540 (PMC8923667; doi:10.7554/eLife.75540)
Supplement: Supplementary file 1. — The areas in Figure 1 were defined based on the Cortical Hierarchy Atlas of the Rhesus Macaque (CHARM) (Jung et al., 2021) and Subcortical Atlas of the Rhesus Macaque (SARM) (Hartig et al., 2021) to closely match the corresponding electrophysiological recording sites. For some of the cortical regions of interest, the CHARM parcellation did not match the recording sites. As a result, the areas were defined according to the respective descriptions (Note: this is the case whenever “Custom” is indicated in the table). Abbreviations: S1/S2 (primary/secondary somatosensory cortex), MT (middle temporal area), LIP (lateral intraparietal area), OFC (orbitofrontal cortex), LPFC (lateral prefrontal cortex), ACC (anterior cingulate cortex), PMd (dorsal premotor cortex), LOFC (lateral OFC), DLPFC (dorso-lateral PFC), VLPFC (ventro-lateral PFC), PFp (polar prefrontal cortex), vmPFC (ventro-medial PFC), sgACC (subgenual ACC), pgACC (pregenual ACC), dACC (dorsal ACC), GPe (external globus pallidus), STN (subthalamic nucleus). [file elife-75540-supp1.docx]

| **Hierarchy** | **Area** | **Level** | **Code** |
| --- | --- | --- | --- |
| Murray et al., 2014 | OFC | 3 | 25 |
|  | ACC | 3 | 3 |
|  | LPFC | 2 | 50 |
|  | S1 | 3 | 92 |
|  | S2 | 3 | 95 |
|  | LIP | 5 | 115 |
|  | MT | 5 | 232 |
| Fascianelli et al. 2019; and Cirillo et al., 2018 | PFo | 3 | 25 |
|  | DLPFC | 6 | 63, 67, 68 |
|  | PFp | Custom | Custom |
|  | PMd | 5 | 81 |
| Cavanagh et al., 2018 | DLPFC | 6 | 63, 67, 68 |
|  | VLPFC | 6 | 53, 70, 74, 75 |
|  | ACC | 6 | 10, 62 |
| Maisson et al., 2021 | vmPFC | 5 | 22 |
|  | pgACC | 6 | 4 |
|  | sgACC | 6 | 5 |
|  | dACC | Custom | Custom |
| Nougaret et al., 2021 | Striatum | 3 | 43 |
|  | STN | 5 | 91 |
|  | GPe | 6 | 56 |
