# Supplementary material for: Intrinsic timescales as an organizational principle of neural processing across the whole rhesus macaque brain
Source: eLife. 2022 Mar 2;11:e75540. doi: 10.7554/eLife.75540 (PMC8923667; doi:10.7554/eLife.75540)
Supplement: Supplementary file 2. — The areas in Figure 2 were defined based on the Cortical Hierarchy Atlas of the Rhesus Macaque (CHARM) (Jung et al., 2021) to match the hierarchies described by Kravitz et al., 2011. Abbreviations: DLPFC (dorso-lateral prefrontal cortex), VIP (ventral intraparietal area), MIP (medial intraparietal area), rIPL (rostral inferior parietal lobule), MT (middle temporal area), MST (medial superior temporal area), LIP (lateral intraparietal area), A46 (area 46), A8A (area 8 A), PMd (dorsal premotor cortex), PMv (ventral premotor cortex). [file elife-75540-supp2.docx]

| **Hierarchy** | **Area** | **Level** | **Code** |
| --- | --- | --- | --- |
| Original Dorsal Pathway | V1 | 3 | 246 |
|  | V2/3 | 3 | 237 |
|  | PG | 6 | 122 |
|  | DLPFC | 3 | 54 |
| Occipito-parietal pathway | V1 | 3 | 246 |
|  | V2 | 4 | 243 |
|  | V3 | 4 | 238 |
|  | V6 | 4 | 98 |
| Parieto-premotor pathway | V6A | 4 | 99 |
|  | MIP | 5 | 108 |
|  | PMd | 5 | 81 |
|  | PMv | 5 | 84 |
|  | VIP | 6 | 110 |
|  | rIPL | 6 | 123 |
| Parieto-prefrontal pathway | MT | 6 | 232 |
|  | MST | 6 | 119 |
|  | 8A | 5 | 51 |
|  | 46 | 5 | 63, 66 |
